# PROTOCOL: Effectiveness of behavioral interventions for smoking cessation among homeless persons: A systematic review and meta‐analysis

**DOI:** 10.1002/cl2.1416

**Published:** 2024-06-14

**Authors:** Runjing Dai, Tiantian Feng, Xiaoting Ma, Juan Cao, Kehu Yang, Jingchun Fan

**Affiliations:** ^1^ Hospital Infection‐Control Department Xi'an Aerospace General Hospital Xi'an Shaanxi P.R. China; ^2^ School of Public Health, Center for Laboratory and Simulation Training, Center for Evidence‐Based Medicine Gansu University of Chinese Medicine Lanzhou Gansu China; ^3^ School of Nursing Gansu University of Chinese Medicine Lanzhou Gansu China; ^4^ Department of Public Health Affiliated Hospital of Gansu University of Chinese Medicine Lanzhou China; ^5^ School of Public Health, Evidence Based Social Science Research Center Lanzhou University Lanzhou China

**Keywords:** behavioral intervention, homeless, meta‐analysis, smoking cessation

## Abstract

This is the protocol for an updated Campbell systematic review. The objectives are as follows: To evaluate the effect of behavioral interventions on smoking cessation among homeless individuals.

## 1 INTRODUCTION

Homelessness is not solely defined by the absence of living spaces; its definition varies depending on the context but can be roughly understood as a life without a long‐term safe place to live (Mabhala et al., 2017). This encompasses staying in short‐term accommodation with friends or relatives, temporary housing in state or charitable institutions (e.g., youth hostels, night shelters), and resorting to sleeping outdoors or in premises not intended or suitable for rent. Simultaneously, it represents a complex social and public health issue (Mabhala et al., 2017; Soar et al., 2020).

Smoking is a prevalent high‐risk behavior among homeless individuals (Chen et al., 2016), with over 70% of adult homeless people being smokers (Baggett et al., 2013). Tobacco‐induced diseases stand as major contributors to morbidity and mortality in this demographic, with the incidence of tobacco‐related chronic diseases being three times higher in adults who have experienced homelessness compared to non‐homeless adults of the same age. This underscores the urgent need for interventions aimed at reducing the burden of tobacco use in this population.

Quitting smoking can significantly reduce mortality and morbidity from smoking‐related diseases and lead to substantial economic savings in healthcare costs (Doll et al., 2004). Additionally, it can improve mental health, reduce stress, and enhance overall quality of life and well‐being (Bloom et al., 2017; Lasser et al., 2000; Parrott, 1995).

Among many smokers, the desire to quit is prevalent, yet accomplishing this feat is challenging (Chaiton et al., 2016). There is evidence suggesting that behavioral therapies can assist individuals in smoking cessation, either in conjunction with or independently of smoking cessation medications (Cahill et al., 2013; Hartmann‐Boyce et al., 2014). Studies have indicated that numerous people utilize behavioral interventions to aid in smoking cessation; however, the content and effectiveness of these interventions may vary significantly (Hartmann‐Boyce et al., 2021).

Baggett et al. (2018, 2019) and Rash et al. (2018) have conducted studies on various behavioral therapies, such as financial incentives, text‐messaging interventions, and contingency management (CM), either as primary interventions or complementary measures. However, the results are inconsistent; while Baggett et al. suggest that financial incentives are more effective, Rash et al. argue that CM yields better smoking cessation outcomes. Bryant et al. (2011) assessed the methodological quality and effectiveness of behavioral smoking cessation interventions across six vulnerable groups, including the homeless, prisoners, indigenous populations, at‐risk youth, individuals with low socioeconomic status, and people with mental illness. Gentry et al. (2019) evaluated the effectiveness of e‐cigarettes for smoking cessation among vulnerable groups, indicating promising results for the use of behavioral intervention measures among certain socially disadvantaged groups.

Soar et al. (2020) endeavored to estimate smoking cessation rates among homeless populations through systematic reviews, but only simple descriptive analyses were conducted due to limited data. Huynh et al. (2022) evaluated the impact of tobacco dependence management in low socioeconomic populations, revealing that the multicomponent intervention achieved a higher smoking cessation rate than the control group; however, no specific intervention was identified. While most of these reviews suggest the effectiveness of these interventions, few studies have directly compared different forms of behavioral intervention.

Moreover, we have conducted meta‐analyses on the effects of various smoking cessation methods in the general population (Dai et al., 2021). Hersi et al. examined diverse smoking cessation interventions across different populations (Chai et al., 2019; Hersi et al., 2019). However, there is a dearth of smoking cessation studies specifically targeting homeless individuals, including investigations into the correlation between homelessness and smoking, as well as evidence‐based smoking cessation programs tailored to the needs of this demographic (Thompson & Hasin, 2011; Wenzel et al., 2010).

Homeless individuals encounter numerous obstacles to quitting, such as the desire to smoke and a lack of social and economic support (Baggett et al., 2019; Pinsker et al., 2018). Additionally, many researchers exclude the homeless from clinical trials for practical reasons (Bandura, 1977; Davis et al., 2002; Young et al., 2006). Consequently, homeless smokers have largely been overlooked in smoking reduction efforts (Tucker et al., 2020), resulting in a scarcity of evidence to inform health decisions regarding smoking cessation among this population.

Certainly, there are studies specifically targeting smoking cessation among homeless individuals (Okuyemi et al., 2012; Richards et al., 2014). Therefore, it would be beneficial to provide an overview of the evidence to effectively promote the health of the homeless population. In this study, we will analyze the effectiveness of behavioral interventions in facilitating smoking cessation among homeless smokers.

## 2 OBJECTIVES

To evaluate the effect of behavioral interventions on smoking cessation among homeless individuals.

## 3 METHODS

### 3.1 Criteria for inclusion and exclusion of studies

Articles will be searched using a set of search terms specifically targeting behavioral therapy for aiding homeless individuals in smoking cessation. Upon retrieving these articles, we will screen them to identify those that meet the coding criteria. Eligible articles must meet the following criteria.

#### 3.1.1 Types of studies

We will include randomized controlled trials (RCTs) and non‐RCTs evaluating any behavioral interventions aimed at smoking cessation among homeless individuals. Case reports, reviews, abstracts, mechanistic discussions, experience summaries, and other types of research studies will be excluded.

#### 3.1.2 Types of participants

Smokers, defined as individuals aged 18 years or older who have used tobacco products daily for at least 3 weeks (excluding e‐cigarettes), and who are experiencing or at risk of homelessness, constitute the sole population under study. According to relevant research in the UK, homeless individuals primarily fall into the following categories (Jia, 2018):

(1) Street sleepers residing in non‐residential conditions.
(2) Individuals temporarily residing in emergency or transitional accommodations (including guest houses, night shelters, and other similar facilities) without permanent housing.
(3) Those who may face loss of housing upon release from prison or due to other circumstances.
(4) Individuals who are at risk of losing their housing in the short term due to threats of violence or abuse.
(5) People residing in uninhabitable environments (such as overcrowded conditions).

We will exclude users of smokeless tobacco, studies where the primary focus is not relevant to smoking behavior among homeless individuals, and individuals not at risk of homelessness.

#### 3.1.3 Types of interventions

We will include all behavioral interventions for homeless smokers, such as behavior change techniques, behavioral counseling, brief advice, individual or group counseling, mindfulness training, financial incentives, motivational interviewing, telephone consultations, mobile text message interventions, and digital interventions (LeFevre & St Louis, 2022; Roberts et al., 2013).

#### 3.1.4 Types of comparators

Control or comparison conditions may include routine care (such as drug therapy or nicotine replacement therapy), or a “minimal” control (e.g., no treatment or a waiting list control), or a combination of these modalities with another behavioral intervention. Studies involving only two simple behavioral therapies as both interventions and controls will be excluded because the effectiveness of the behavioral intervention cannot be discerned. Additionally, interventions that are not primarily aimed at smoking cessation will be excluded.

#### 3.1.5 Types of outcomes

The primary outcome measure will be the abstinence rate at least 1 month after the initiation of the intervention, with preference given to reported sustained abstinence rates. In cases where sustained abstinence rates are not reported, point abstinence rates will also be considered acceptable. If a study reports abstinence rates at multiple time points, data will be extracted from all time points. If a sufficient number of studies report similar time points, the effects of interventions across multiple time points will be analyzed in a subgroup analysis, including follow‐up times such as post‐treatment, 12, 24, and 52 weeks after treatment.

Additionally, if there are enough studies reporting adverse events, these will be selected as the secondary outcome measure.

#### 3.1.6 Other criteria

We will include all studies that meet the inclusion criteria, even those with incomplete data or unclear outcome measures. However, studies with insufficient data provided by the primary authors to calculate the effect size will be excluded from the meta‐analysis but will still be included in the review. Only articles published in English or Chinese will be considered. There are no limitations regarding the year of publication or publication status. Furthermore, articles with duplicate publications will be excluded.

### 3.2 Search strategy for identification of relevant studies

We will identify published literature by searching the following databases: PubMed, Embase, the Cochrane Central Register of Controlled Trials, Web of Science (including the Science Citation Index databases, Social Sciences Citation Index databases, and Science Citation Index Expanded databases), PsycINFO, CINAHL, and The Campbell Library databases. Additionally, we will systematically search three Chinese databases: the Chinese Biomedical Literature Database, China National Knowledge Infrastructure, and Wan Fang Database. Ongoing or unpublished trials will be searched for in the WHO International Clinical Trials Registry (ICTRP).

We will also search for gray literature, which encompasses non‐publicly published materials such as academic dissertations, unpublished conference proceedings, technical reports, archives, internal journals, and donated materials. This search will be conducted on the Google Scholar website using the search term “smoking cessation, behavioral therapy, homelessness.”

Furthermore, we will perform forward and reverse citation screening by systematically reviewing citations and bibliographies. Any literature not retrieved through the initial searches will be supplemented by examining the references of retrieved articles and reviews.

Our search terms will include “smoking cessation,” “behavior therapy,” and “homeless,” along with multiple synonyms for each term incorporated into the search. The search strategy for PubMed is provided in Table 1.

**Table 1:** Search strategies.

Search strategy for PubMed	
#1	Search: “Smoking Cessation”[Mesh]
#2	Search:((((tobacco[Title/Abstract]) OR (smok*[Title/Abstract])) OR (cigaret*[Title/Abstract])) OR (nicotine[Title/Abstract])) AND (((((((quit*[Title/Abstract]) OR (ceas*[Title/Abstract])) OR (cessation[Title/Abstract])) OR (give up[Title/Abstract])) OR (gave up[Title/Abstract])) OR (giving up[Title/Abstract])) OR (stop*[Title/Abstract]))
#3	#1 OR #2
#4	Search: “Behavior Therapy”[Mesh] OR “Behaviour Therapy”[Mesh]
#5	Search: (((Behavio* Therap*[Title/Abstract]) OR (Conditioning Therap*[Title/Abstract])) OR (Behavio* Change Technique*[Title/Abstract])) OR (Behavio* Modification*[Title/Abstract])
#6	#4 OR #5
#7	Search: “Ill‐Housed Persons”[Mesh] OR “Homeless Youth”[Mesh]Sort by: Most Recent
#8	Search: ((((((insecur*[Title/Abstract]) OR (Ill Housed[Title/Abstract])) OR (Homeless*[Title/Abstract])) OR (Unhous*[Title/Abstract])) OR (Shelterless[Title/Abstract])) OR (Street[Title/Abstract])) AND ((people[Title/Abstract]) OR (person*[Title/Abstract]))
#9	#7 OR #8
#10	#3 AND #6 AND #9

Search strategy for CNKI	
#1	(主题=戒烟) (#1 (topic = smoking cessation))
#2	(主题=行为疗法) OR (主题=矫正疗法) OR (主题=行为治疗)OR (主题=团体治疗) OR (主题=正念疗法) (#2 (topic = behavior therapy) OR (topic = corrective therapy) OR (topic = group therapy) OR (topic = Mindfulness therapy))
#2	(主题=无家可归) OR(主题=流浪汉) OR(主题=流动人口) (#3 (topic = homeless people) OR (topic = vagrant) OR (topic = floating population))
#4	#1 AND #2 AND #3

### 3.3 Study selection

Each retrieved citation will undergo review by two independent reviewers (Runjing Dai and Xiaoting Ma) against the eligibility criteria. The selection process will be divided into two stages.

In the first stage, all retrieved articles will be imported into EndNote X9 software. We will initially identify and remove duplicate documents using the software's “Find Duplicates” function. Subsequently, two independent reviewers will cross‐check to ensure that any remaining duplicate documents are identified and deleted based on basic information such as article title and author.

After excluding duplicates, two reviewers (Runjing Dai and Tiantian Feng) will assess titles and abstracts according to the inclusion and exclusion criteria. By examining the abstracts and titles, the reviewers will determine whether the articles meet the fundamental criteria, including language, study design, population, and intervention, to conduct preliminary screening.

In the second stage, articles that cannot be definitively identified in the first stage will be assessed individually by two reviewers (Runjing Dai and Tiantian Feng). They will review the full text in order of language, study design, population, interventions, outcome measures, decisions, and other relevant information. Articles failing to meet the inclusion criteria will be excluded, while those meeting the criteria will be included.

For studies where only a subset of samples qualifies for inclusion, they will be retained in the review without undergoing meta‐analysis. Any disagreements between reviewers will be resolved through discussion and consultation with a third reviewer (Jingchun Fan).

**Figure 1:**
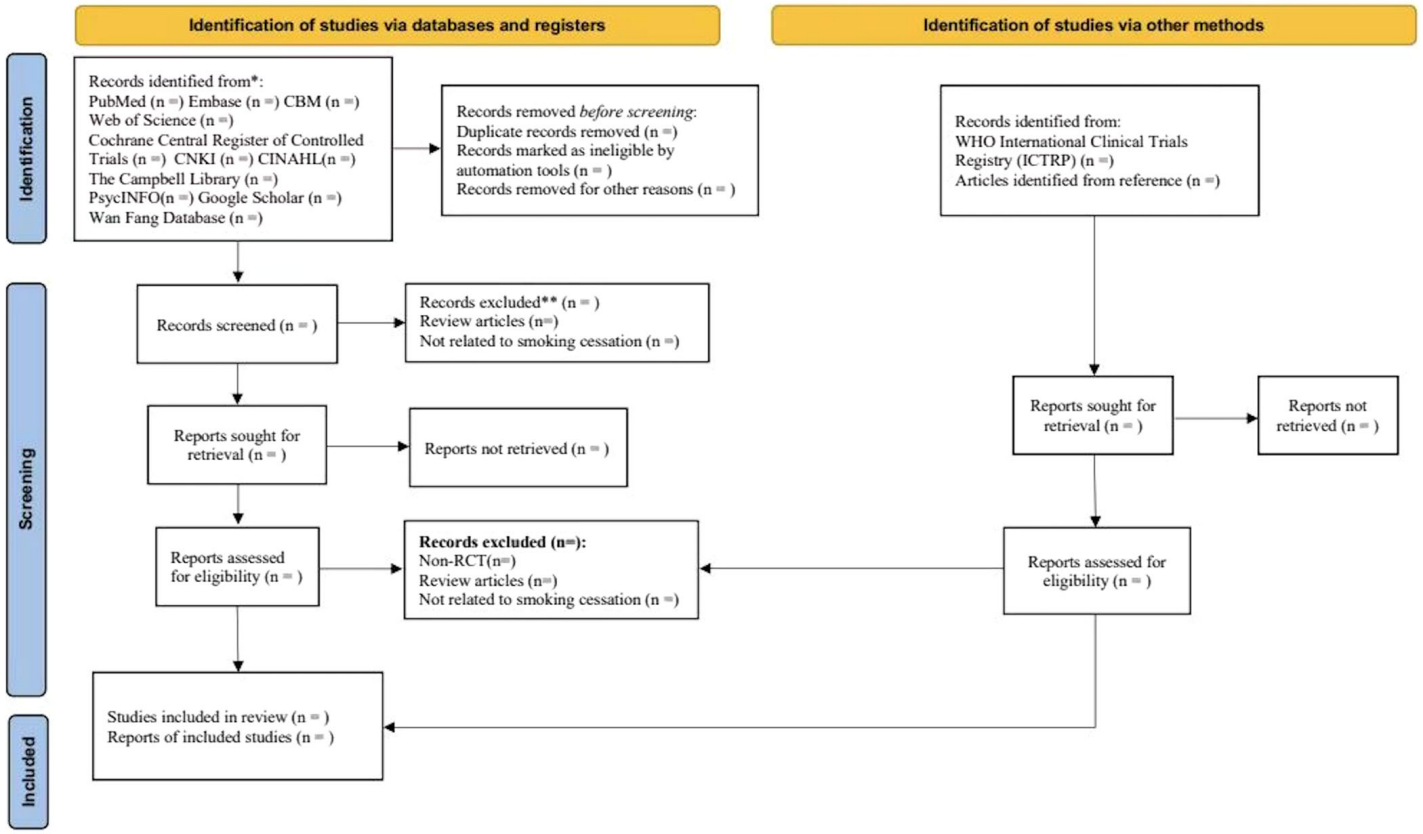
The flow diagram for study screening.

The flow chart illustrating the study selection process is displayed in Figure 1.

### 3.4 Data extraction and management

Two reviewers (Runjing Dai and Juan Cao) will independently extract data from the included studies. Before data extraction, both reviewers participated in calibration exercises where they independently extracted relevant information from the same article to ensure consistency in data extraction.

For data extraction, we will utilize the tools provided by Campbell (Covidence: https://www.covidence.org/reviewers/) to obtain relevant information and data for inclusion in the study. The extracted information will include: Basic bibliographic details (title, first author, publication year, journal, and language); Study characteristics (age, country, total sample size, male sample size, and subject); Intervention details (intervention and control group treatments, duration of intervention, and follow‐up time); Study outcomes (outcomes, measurement methods, effect sizes, and adverse effects).

We will encode the data for each study according to the following rules:

Number randomized to each group; Mean age across the study population; Percentage of men across the study population; Mean cigarettes per day at baseline and deadline; Weeks of treatment; Weeks of follow‐up; Number who quit in each group; Definition of cessation.

If there are studies that use quantitative data (such as cigarettes per day or expiratory CO_2_ levels) to assess smoking cessation, we will convert this into a binary variable based on the definition of smoking cessation provided in each study.

For other quantitative data, we will extract the difference data before and after treatment and the raw data at different times during treatment for analysis. If studies report indicators at the end of treatment, the difference data will be calculated based on the data before and after treatment as reported. During the data coding process, we may encode various forms of data, resulting in the calculation of effect sizes for multiple variable types. For example, binary category data may be calculated as odds ratios (ORs), while continuous variable results may be calculated as average differential effect sizes. If studies report the same indicator but variable types are inconsistent, we will attempt to transform different data into the same variable type whenever possible. For instance, ORs can be converted into Cohen's *d*, and Pearson correlations can also be transformed into Cohen's *d* (Borenstein & Hedges, 2019). Additionally, there may be data where both independent and dependent variables are continuous. These data will be used to calculate product‐moment correlation effect size statistics. In reporting results, we will only compare effect sizes that are similar to each other and combine them within types for proper analyses. If a study included more than two intervention groups, only the intervention and control groups that met the eligibility criteria were included. If necessary, the authors of the original trial will be contacted for further information and clarification of data. Any disagreements will be resolved by agreement or consultation with third party review authors (Jingchun Fan).

### 3.5 Risk of bias assessment

The quality assessment of RCTs will be conducted according to the Cochrane Handbook for Systematic Reviews of Interventions (Higgins et al., 2011). This assessment will include the following domains: random sequence generation, allocation concealment, blinding of participants and personnel, blinding of outcome assessment, incomplete outcome data, selective reporting, and other biases.

Non‐RCTs will be assessed using the ROBINS‐I checklist (Sterne et al., 2016). This checklist primarily includes items related to confounding bias, selection bias, intervention classification bias, deviation from expected intervention bias, missing data bias, outcome measurement bias, and reported outcome selection bias.

Two independent reviewers (Runjing Dai and Tiantian Feng) will assign judgments of low, high, or unknown risk for each risk of bias domain in each study. If necessary, we will contact the authors of included studies to obtain supplementary or clarifying information. The risk of bias for each area of each study will be presented graphically. Any disagreements will be resolved through discussion between the reviewers and, if necessary, consultation with a third reviewer (Jingchun Fan).

### 3.6 Measures of treatment effect

We will use ORs with 95% confidence intervals (CIs) to represent the estimate of the effect for dichotomous data. For continuous data, we will extract the mean and standard deviation with 95% CI of the relevant results. Alternatively, we will extract other relevant data that can be converted into Cohen's *d* (e.g., *F* value, *t* value, *p* value).

If the same outcome is measured in different ways, the intervention effect size will be represented by a normalized average with a 95% CI. If multiple effect sizes are provided from the same study and there is a dependency between the effects, we will address this dependency using a three‐level meta‐analytic model. This model accommodates variations in the number of effect sizes per study, variance between studies, correlation between pairs of results, and sample size of studies, thereby enhancing the study methodology (van den Noortgate et al., 2014).

### 3.7 Dealing with missing data

We will make every effort to contact the study authors to obtain missing data. Studies with incomplete data will be excluded from the meta‐analysis but will still be included in the review.

### 3.8 Assessment of heterogeneity

Heterogeneity will be assessed using *Q* statistics and *I*² statistics. The *I*² test will estimate the degree of heterogeneity, with an *I*² value between 30% and 50% considered indicative of moderate heterogeneity, while a value between 50% and 90% will be indicative of severe heterogeneity. Potential clinical heterogeneity will be assessed through regression analysis.

### 3.9 Data synthesis

We will combine the results of multiple trials that examine similar interventions with comparable methods in the same population to estimate pooled intervention effects through meta‐analysis. For comprehensive effect model selection, we will choose the random‐effects model. Data analysis will be conducted using Review Manager V.5.3 software and Stata 15.

If meta‐analysis between studies is not feasible due to limited literature or other reasons, we will provide a narrative summary of the results from the individual studies.

### 3.10 Assessment of publication bias

When a meta‐analysis includes 10 or more RCTs, we will visually assess the potential small study effect using funnel plot asymmetry. We will select either Begg's or Egger's test to quantify any asymmetry attributed to publication bias.

### 3.11 Subgroup analysis and investigation of heterogeneity

When sufficient data are available, subgroup analysis will be conducted based on age, smoking volume, duration/frequency of treatment, type of control intervention, and the initial physical condition of the participants. For example, age‐based subgroup analysis will categorize participants into young adults (18–45 years old), middle‐aged (46–68 years old), and elderly (69 years and older). Alternatively, participants will be categorized based on their physical condition before receiving the first treatment, dividing them into general smokers, smokers with underlying cardiovascular diseases, smokers with respiratory diseases, and smokers with combined alcohol dependence.

Additionally, subgroup analysis will be conducted based on multiple time points, such as after treatment, 12, 24, and 52 weeks after treatment, according to the subjects' follow‐up time. Simultaneously, subgroup analysis will also be performed based on the type of intervention. For instance, if the control group intervention includes medication and behavioral support for smoking cessation, subgroup analysis will be conducted accordingly.

### 3.12 Sensitivity analysis

Sensitivity analysis, using the “leave‐one‐out” method, will be conducted. This involves performing meta‐analysis after excluding one or several studies to observe whether the combined analysis results and heterogeneity change. This allows us to evaluate the stability of the meta‐analysis results.

### 3.13 Summary of findings and assessment of the certainty of the evidence

We will use the Grades of Recommendation, Assessment, Development, and Evaluation Working Group (GRADE) handbook to assess the quality of evidence for each main outcome (Schunemann et al., 2011). RevMan will support a “Summary of Findings” table containing information on interventions, controls, outcomes, and more. The quality of evidence will be categorized into three levels: high, moderate, or low.

The above tasks will be performed independently by two reviewers. In case of disagreement, a decision will be reached through consultation with a third investigator.

## TIMEFRAME

We have commenced collecting data from eight electronic databases for published studies dating back to their inception. Following the completion of the search for published studies, we will commence the search for new unpublished studies. Our aim is to compile a comprehensive bibliography of both published and unpublished studies by July 2023.

Upon completion of the study search, we will proceed to assess the eligibility of the articles. We anticipate completing this process by the end of September 2023. Once we have finalized the list of eligible articles, we will begin data coding from the selected studies and subsequently calculate effect sizes where feasible. We expect to complete the calculation of effect sizes by October 2023, after which we will commence manuscript writing. Our target is to submit the written report to the Campbell Collaboration Group by December 2023.

## PLANS FOR UPDATING THE REVIEW

Once we submit a written report to the Campbell Collaboration and publish a paper to a journal, we will begin work on updating the review. We intend to revise the Campbell's Collaboration review every 3 years. We will provide the date of any amendment, a description of the change and the reason for the amendment to the protocol.

## PATIENT CONSENT

Not required.

## DECLARATIONS OF INTEREST

All authors declare no potential interest.

## CONTRIBUTIONS OF AUTHORS

Runjing Dai and Tiantian Feng drafted the protocol, and all authors reviewed the draft and approved the final version.

## ETHICS AND DISSEMINATION

There are no research ethical issues because this is a systematic review of published literature. The results of this study will be published in a peer‐reviewed journal. We will optimize search engine visibility by using keywords such as “homeless,” “smoking cessation,” and others in the titles to facilitate search engine identification of relevant papers. Full‐text pre‐publication versions of papers will be deposited in institutional repositories as permitted by copyright rules.

Upon publication, we will disseminate this information through social media platforms such as WeChat, Twitter, and Facebook to reach individuals who may not typically engage in literature research on smoking cessation among homeless individuals. Additionally, we will submit our findings to public health policymakers, including the National Health Commission of the People's Republic of China, the Chinese Center for Disease Control and Prevention, and other relevant institutions, to provide a scientific basis for tobacco control policy formulation.
